# Expression of GARP Is Increased in Tumor-Infiltrating Regulatory T Cells and Is Correlated to Clinicopathology of Lung Cancer Patients

**DOI:** 10.3389/fimmu.2017.00138

**Published:** 2017-02-14

**Authors:** Hao Jin, Liping Sun, Lu Tang, Wenwen Yu, Hui Li

**Affiliations:** ^1^Department of Immunology, Tianjin Medical University Cancer Institute and Hospital, Tianjin, China; ^2^Key Laboratory of Cancer Immunology and Biotherapy, Tianjin, China; ^3^National Clinical Research Center of Cancer, Tianjin, China; ^4^Division of Rheumatology, Tianjin First Center Hospital, Tianjin, China; ^5^Department of Gastrointestinal Cancer Biology, Tianjin Medical University Cancer Institute and Hospital, Tianjin, China

**Keywords:** lung cancer, regulatory T cells, GARP, TGF-β, Foxp3, LAP, clinicopathology

## Abstract

Regulatory T cells (Tregs) are immunosuppressive T cells that play an important role in immune homeostasis. Multiple markers have been associated with the characterization, as well as function of Tregs. Recently, glycoprotein A repetitions predominant (GARP), a transmembrane protein containing leucine-rich repeats, has been found to be highly expressed on the surface of activated Tregs. GARP maintains Tregs’ regulatory function and homeostasis through the activation and secretion of transforming growth factor β. In this study, we investigated the expression of GARP in Tregs from the peripheral blood (PB) and tumor tissues of lung cancer patients. The association between the proportion and expression level of GARP on Tregs and the clinicopathological factors of lung cancer patients was also analyzed. Results showed that in the tumor tissues of patients with lung cancer, GARP expression was increased in Tregs and was associated with lymph node metastasis, distant metastasis, and clinical stage. Furthermore, the infiltrating Tregs from early stage patients exhibited higher GARP expression than that from advanced cancer patients, which indicated that GARP might be an early prognostic biomarker. *In vitro* coculture studies demonstrated that human lung cancer cell lines might induce the expression of GARP in Tregs by certain mechanisms. Overall, this research demonstrated the potential value of GARP in Tregs definition and cancer immunotherapy.

## Introduction

For years, accumulating evidence has indicated that tumor microenvironment plays an important role in tumor progression, invasion, and metastasis (1). The tumor microenvironment is a complex system consisting of cells, soluble factors, signaling molecules, extracellular matrix, and mechanical cues (2). Tumors possess infiltrating cells of both innate and acquired immunity, such as myeloid-derived suppressor cells, macrophages, dendritic cells, mast cells, eosinophils, neutrophils, NK cells, and lymphocytes. These cells coordinately form a complex regulatory network, which fosters tumor growth by creating an environment that enables cancer to evade immune surveillance and destruction (3). Regulatory T cells (Tregs), as the primary immunosuppressive cells, can inhibit the antitumor immune response and promote tumor progression. A number of studies have provided clear evidence that the number of tumor-infiltrating Tregs was increased in multiple tumors, such as renal carcinoma, gastrointestinal cancer, breast carcinoma, lung cancer, prostatic carcinoma, melanoma, and ovarian carcinoma (4–10).

The immunosuppressive activity of Tregs makes it one of the effective targets of immunotherapy, but its application is limited by the lack of specific markers. To date, the transcription factor, Foxp3, which is necessary for the development and function of Tregs, is the most specific marker for Tregs (11). The depletion or mutation of Foxp3 in Tregs leads to common autoimmune and inflammatory diseases such as IPEX, which is characterized by immune dysfunction, polyendocrinopathy enteropathy, and X-linked syndrome (12, 13). However, Foxp3 cannot be used to isolate functional Tregs for further study because it is located in the nucleus, and it can also be expressed in conventional T cells (14–16). Although a number of surface and intracellular molecules are highly expressed in Tregs, none of them is a reliable and suitable molecule for functional Tregs isolation, let alone therapeutic applications (17, 18). Therefore, a novel marker that may identify a special subset of Tregs will provide a new method for the research of Tregs.

Glycoprotein A repetitions predominant (GARP), a transmembrane protein consisting of 662 amino acids, is highly expressed on the surface of a certain subset of Tregs and is activated through TCR stimulation (19). GARP increases the suppressive function of Tregs and promotes secretion and activation of transforming growth factor β (TGF-β), which participates in Tregs’ function and homeostasis (20–23). GARP can competitively bind latent TGF-β, a complex formed by mature TGF-β and latency-associated peptide (LAP), through its horseshoe-shaped solenoid ectodomain (21, 24, 25). As a result, GARP^+^ Tregs became the storage of TGF-β, and this T cell subset possesses strong immunosuppressive effect. However, the exact role of GARP^+^ Tregs in tumor microenvironment remains little understood.

In this study, we analyzed the expression of GARP on Tregs and Tconvs in peripheral blood (PB) and tumor tissue from lung cancer patients. Our data suggested that GARP expression was increased in the Tregs from tumor tissues of patients with lung cancer and the proportion of GARP-expressing Tregs was obviously higher in lung cancer patients without lymph node metastasis or distant metastasis and in patients with early clinical stage. In addition, we demonstrated that lung cancer cell lines could induce GARP expression in Tregs in *in vitro* coculture assays. Our data indicated that GARP is a specific marker for identifying a subset of Tregs that is activated and highly immunosuppressive. Therefore, GARP^+^ Tregs may be a potential target for cancer immunotherapy.

## Materials and Methods

### Healthy Donors and Patients

Blood samples were obtained peripherally from 50 first-time admitted lung cancer patients in Tianjin Medical University Cancer Institute and Hospital (Tianjin, China) and from 10 healthy donors, after receiving written informed consent. Tumor tissues were obtained during the surgery from 39 first-time admitted lung cancer patients in Tianjin Medical University Cancer Institute and Hospital (Tianjin, China), after receiving written informed consent. The procedure used was approved by the Ethics Committee of the Tianjin Medical University Cancer Institute and Hospital. None of the patient received surgery, radiotherapy, chemotherapy, or other medical intervention before the blood collection or the surgery. The characteristics of the study subjects are summarized in Tables 1 and 2.

**Table 1:** **Correlation between clinicopathological characteristics and frequency of Tregs, glycoprotein A repetitions predominant (GARP) expression on Tregs in tumor tissues from lung cancer patients**.

	Cases	Foxp3^**+**^ Tregs/CD4^**+**^ T cells (%)	*P* value	GARP^**+**^/Foxp3^**+**^ Tregs (%)	*P* value	GARP mean fluorescence intensity, median	*P* value
**Gender**							
Male	27	8.740 (5.040, 15.20)	0.0513	8.660 (3.130, 27.20)	0.3268	2,076 ± 1,083	0.2716
Female	12	5.185 (3.810, 6.398)		10.65 (5.940, 41.28)		2,503 ± 1,149	
**Age (year)**							
≤60	16	7.220 (3.873, 13.28)	0.6737	8.875 (4.165, 29.83)	0.9833	2,253 ± 1,427	0.8345
>60	23	6.400 (5.040, 11.20)		9.270 (3.130, 27.20)		2,176 ± 852.3	
**Smoke**							
No	15	5.170 (4.380, 8.620)	0.0902	15.00 (6.420, 35.70)	0.1380	2,658 ± 1,224	0.2408
Yes	24	9.005 (4.940, 14.73)		7.045 (3.123, 23.10)		2,176 ± 1,230	
**Histology**							
Adenocarcinoma	26	7.510 (4.788, 13.23)	0.9986	8.875 (4.733, 35.88)	0.6470	2,261 ± 1,227	0.6692
Squamous cell carcinoma	9	6.430 (4.310, 15.90)		9.270 (2.640, 18.50)		2,266 ± 922.5	
Others	4	6.075 (4.750, 21.14)		9.415 (1.738, 47.85)		1,728 ± 594.1	
**Lymphatic invasion**							
Absent	24	6.350 (4.483, 13.78)	0.9999	12.90 (4.090, 52.13)	0.0465	2,524 ± 1,171	0.0221
Present	15	6.430 (4.780, 13.20)		7.470 (2.920, 10.10)		1,702 ± 793.4	
**Distant metastasis**							
Absent	34	6.280 (4.680, 11.70)	0.1074	10.30 (5.005, 35.88)	0.0001	2,398 ± 1,023	0.0038
Present	5	11.30 (6.815, 31.30)		2.250 (0.9070, 3.110)		916.2 ± 779.2	
**Clinical stage**							
I + II	22	6.415 (4.688, 14.75)	0.7602	22.00 (8.423, 55.28)	0.0001	2,681 ± 1,094	0.0014
III + IV	17	5.650 (4.510, 12.25)		4.590 (2.325, 8.275)		1,595 ± 796.1	

**Table 2:** **Correlation between clinicopathological characteristics and frequency of Tregs, glycoprotein A repetitions predominant (GARP) expression on Tregs in peripheral bloods from lung cancer patients**.

	Cases	Foxp3^**+**^ Tregs/CD4^**+**^ T cells (%)	*P* value	GARP^**+**^/Foxp3^**+**^ Tregs (%)	*P* value	GARP mean fluorescence intensity	*P* value
**Gender**							
Male	34	6.010 (4.695, 7.608)	0.5637	0.3175 (0.0, 0.6118)	0.2544	444.5 (0.0, 548.0)	0.5508
Female	16	5.655 (3.068, 6.828)		0.4920 (0.1908, 0.8838)		407.5 (312.3, 659.5)	
**Age (year)**							
≤60	24	5.950 (5.030, 7.603)	0.2410	0.2085 (0.0, 0.7090)	0.2003	364.5 (0.0, 568.8)	0.3239
>60	26	5.545 (2.948, 6.948)		0.4590 (0.1948, 0.8440)		467.5 (295.0, 555.0)	
**Smoke**							
No	20	5.100 (2.340, 7.043)	0.0631	0.5595 (0.01775, 1.205)	0.2129	446.0 (270.3, 554.3)	0.8861
Yes	30	6.150 (4.900, 7.790)		0.3175 (0.0855, 0.5407)		407.5 (0.0, 601.3)	
**Histology**							
Adenocarcinoma	33	5.830 (4.510, 7.555)	0.8211	0.3670 (0.0925, 0.7395)	0.2484	435.0 (240.0, 565.5)	0.2484
Squamous cell carcinoma	15	5.020 (3.050, 7.580)		0.3310 (0.0, 1.060)		385.0 (0.0, 511.0)	
Others	2	6.015 (5.950, 6.080)		1.094 (0.1670, 2.020)		570.0 (540.0, 600.0)	
**Lymphatic invasion**							
Absent	24	5.240 (3.053, 6.925)	0.2566	0.2685 (0.0, 0.7615)	0.3793	407.5 (0.0, 535.5)	0.6802
Present	26	6.375 (4.790, 7.588)		0.4935 (0.1538, 0.7645)		448.0 (258.0, 579.0)	
**Distant metastasis**							
Absent	42	5.450 (4.343, 6.848)	0.0737	0.3175 (0.0, 0.7405)	0.3021	413.0 (0.0, 622.3)	0.8494
Present	8	7.995 (3.798, 9.090)		0.5265 (0.2580, 1.117)		421.5 (254.8, 489.0)	
**Clinical stage**							
I + II	22	5.080 (4.080, 6.265)	0.1153	0.2245 (0.0, 0.7405)	0.2974	467.5 (0.0, 690.0)	0.5352
III + IV	28	6.825 (4.955, 7.670)		0.4935 (0.1340, 0.7955)		407.5 (222.0, 507.5)	

### Isolation of Mononuclear Cells

Peripheral venous blood was drawn and collected into tubes containing EDTA-K2. The blood was centrifuged with lymphoprep (Axis-shield, Oslo, Norway), and PBMCs were collected at the interface and washed with PBS. The tumor tissue was grinded into single-cell suspension and centrifuged with lymphoprep to remove cell debris, and mononuclear cells were collected at the interface and washed with PBS.

### Flow Cytometry and Antibodies

After blocking FcR, cells were incubated with appropriately diluted antibodies and washed with PBS. For intracellular staining of Foxp3, cells were fixed and permeabilized using a Fix/Permeabilization Kit (eBioscience, San Diego, CA, USA) and stained with antihuman Foxp3-APC mAb (BD Biosciences, San Diego, CA, USA, clone 259D/C7, cat num 560045). Acquisition was performed using FACSCanto II equipped with FACSDiva Version 6.1.3 (BD Biosciences). Data analysis was conducted using FlowJo Version 7.6.2 Software (Tree Star, Ashland, OR, USA). The antibodies used for surface staining in this study included antihuman CD4-PerCP/Cy5.5 (BD Bioscience, San Diego, CA, USA, clone RPA-T4, cat num 560650), antihuman GARP-PE (Miltenyi Biotec, Germany, cat num 130-103-889), and antihuman LAP (TGF-β)-APC (R&D, USA, cat num FAB2463A). The antibody used for intracellular staining included antihuman Foxp3-APC.

### Cell Isolation and *In Vitro* Cell Culture

CD4^+^CD25^+^ Tregs were purified from freshly isolated human PBMCs using human CD4^+^CD25^+^ Treg isolation kit, LD and MS column (Miltenyi Biotec, Germany).

For *in vitro* coculture assays, CD4^+^CD25^+^ Tregs were cultured alone or cocultured for 72 h with lung cancer cell lines H460, LTEP-A-2, GLC-82, A549, and H520 at a ratio of 10:1 and seeded in a 24-well plate in RPMI 1640 medium containing 10% FBS as well as 300 U/ml IL-2. The negative controls were the Tregs cultured alone whereas the positive controls were the cells stimulated with Dynabeads^®^ Human T-Activator CD3/CD28 (Life Technologies AS). For *in vitro* transwell coculture assays, lung cancer cell lines were added into a 24-well plate in the medium described above, then Tregs were seeded into 0.4-µm transwell inserts (Corning Life Sciences) in each well for 72 h culturing. For supernatant culture assays, cell supernatants of A549 and H520 were collected after these two cancer cells were cultured alone for 72 h. Then Tregs were cultured in the conditioned medium, which was composed by supernatant and normal medium at a ratio of 1:3 for 72 h. All the *in vitro* experiments were repeated for three times.

### Statistical Analysis

All statistical analysis was performed with SPSS Statistics 19 (IBM Corporation, NY, USA). Numerical data were expressed as the mean ± SD when the Kolmogorov–Smirnov test revealed a normal distribution of these data. Comparisons of numerical data were performed by two-sample *t*-test, independent sample *t*-test, or one-way ANOVA test. The non-parametric *t*-test and Kruskal–Wallis test were used to determine the statistical significance when the distribution agreed with the non-normal distribution, and the data are shown as the median and interquartile range. *P* < 0.05 was considered statistically significant.

## Results

### GARP Was Mainly Expressed in Tumor-Infiltrating Foxp3^+^ Tregs in Lung Cancer Patients

**Figure 1:**
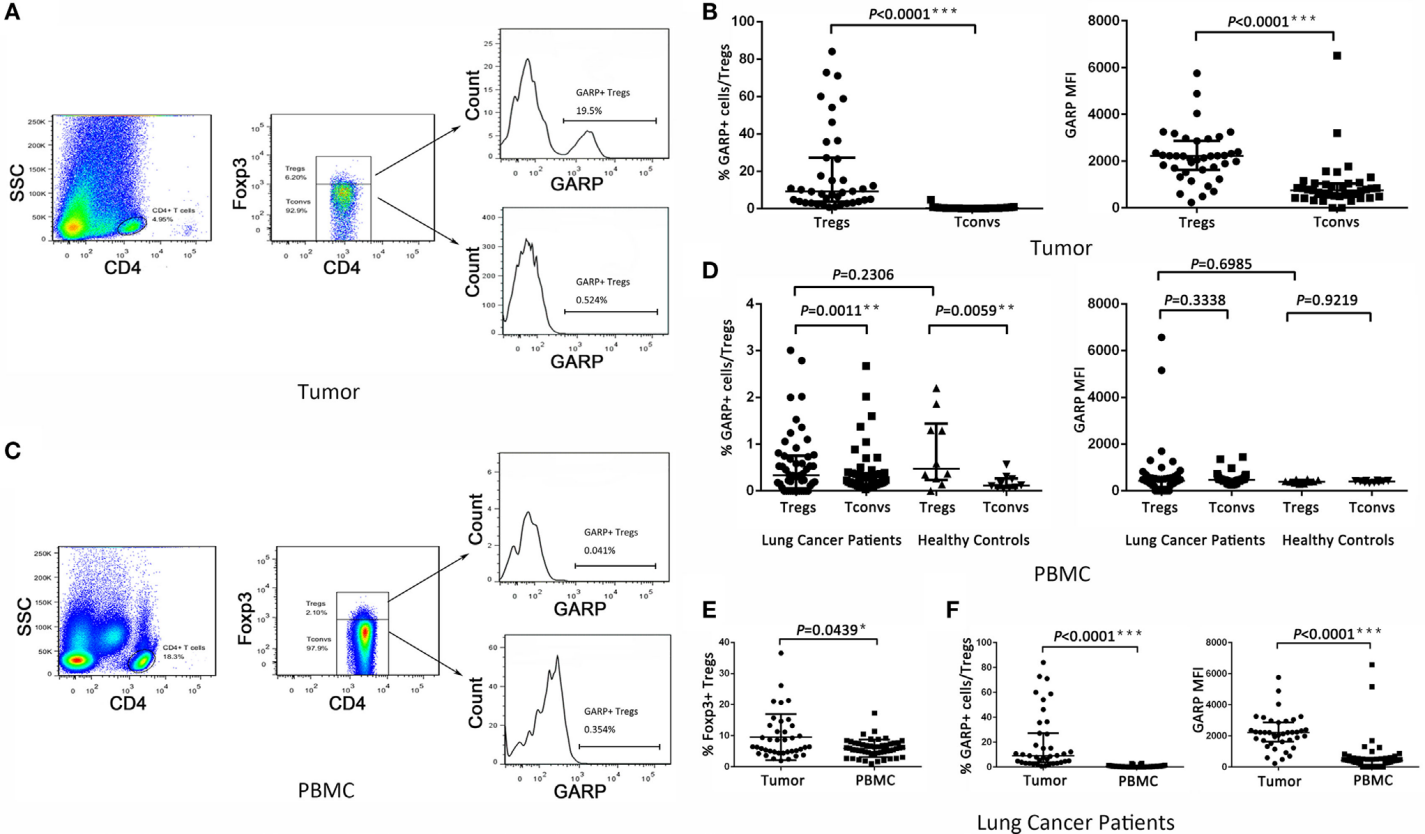
**Level of glycoprotein A repetitions predominant (GARP) expression on Tregs and Tconvs from lung cancer patients**. Mononuclear cells from tumor tissues and peripheral bloods (PBs) from the indicated sources were stained for CD4, GARP, and intracellular Foxp3 and analyzed with FACS. **(A,C)** Proportion of GARP^+^ Tregs in tumor tissues and PBs from lung cancer patients or healthy donors, respectively. Typical flow plots are shown. **(B)** Proportion of GARP^+^ Tregs and mean fluorescence intensity (MFI) of GARP expression by Tregs and Tconvs in tumor tissues from lung cancer patients. Data shown are the summary from 39 lung cancer patients. **(D)** Proportion of GARP^+^ Tregs and MFI of GARP expression by Tregs and Tconvs in PBs both from lung cancer patients and healthy donors. Data shown are the summary from 50 lung cancer patients and 10 healthy donors. **(E)** Proportion of Foxp3^+^ Tregs from lung cancer patients. Data shown are the summary from 39 lung cancer patients’ tumor tissues and 50 lung cancer patients’ PBs. **(F)** Proportion of GARP^+^ Tregs and MFI of GARP expression from lung cancer patients. Data shown are the summary from 39 lung cancer patients’ tumor tissues and 50 lung cancer patients’ PBs. Results are expressed as median and interquartile range. *P* value shown is obtained from the comparison between the indicated groups by non-parametric *t*-test and Kruskal–Wallis test.

We first assayed the expression of GARP in Foxp3^+^ Tregs and Foxp3^−^ Tconvs from 39 tumor tissues in lung cancer patients by flowcytometry (Figure 1A). We found that, in tumor tissues, the proportion of GARP^+^ cells in Foxp3^+^ Tregs [9.090% (3.830%, 27.20%)] was remarkably higher than that in Foxp3^−^ Tconvs [0.2410% (0.1440%, 0.4610%), *P* < 0.0001] (Figure 1B). The level of GARP expression on per cell basis, e.g., the mean fluorescence intensity (MFI), was also examined. The MFI of GARP expression by Foxp3^+^ Tregs [2216 (1628, 2863)] was almost twofold greater than that by Foxp3^−^ Tconvs [752.0 (483.0, 1040), *P* < 0.0001] from tumor tissues (Figure 1B). These data suggested that the expression of GARP in Foxp3^+^ Tregs was markedly higher than that in Foxp3^−^ Tconvs from tumor tissues. In contrast, we detected GARP expression in Foxp3^+^ Tregs and Foxp3^−^ Tconvs from 50 PBs in lung cancer patients and 10 PBs in healthy donors (Figure 1C). As a result, the proportion of GARP^+^ cell in Foxp3^+^ Tregs from lung cancer patients was almost similar with that from healthy donors [0.3390% (0.05325%, 0.7548%) vs. 0.4780% (0.2353%, 1.440%), *P* = 0.2306], while both of them were a very little bit higher than that in Foxp3^−^ Tconvs from lung cancer patients [0.2315% (0.1368%, 0.3825%), *P* = 0.0011] and healthy donors [0.1155% (0.06775%, 0.2648%), *P* = 0.0059]. The MFI of GARP in Foxp3^+^ Tregs from lung cancer patients was also similar with that from the healthy controls [420.0 (153.0, 562.3) vs. 384.0 (332.8, 461.8), *P* = 0.6985], but compared with the proportion of GARP^+^ cell, the MFI of GARP in Foxp3^+^ Tregs was similar with that in Foxp3^−^ Tconvs [420.0 (153.0, 562.3) vs. 463.0 (307.5, 494.5), *P* = 0.3338; 384.0 (332.8, 461.8) vs. 404.0 (354.8, 429.8), *P* = 0.9219] (Figure 1D). These indicated that GARP expression remained in a low level in Foxp3^+^ Tregs both from lung cancer patients and healthy donors.

We compared the GARP expression in Foxp3^+^ Tregs between tumor tissues and PBs in lung cancer patients. The proportion of GARP^+^ cells in Foxp3^+^ Tregs from tumor tissues [9.090% (3.830%, 27.20%)] was markedly higher than that from PBs [0.3390% (0.05325%, 0.7548%), *P* < 0.0001], and the MFI of GARP expression by Foxp3^+^ Tregs from tumor tissues was more than five times that from PBs [2216 (1628, 2863) vs. 420.0 (153.0, 562.3), *P* < 0.0001] whereas the proportion of Foxp3^+^ Tregs in CD4^+^ T cells from tumor tissues [6.400% (4.780%, 13.20%)] was also higher than that from PBs [5.890% (4.343%, 7.520%), *P* = 0.0439] (Figures 1E,F). Thus, GARP was mainly expressed in tumor-infiltrating Foxp3^+^ Tregs instead of that from lung cancer patients PBs.

### GARP Expression on Tregs Is Highly Associated with the Clinicopathological Characteristics of Lung Cancer Patients

As a result of their superior suppressive function, GARP expression on Tregs may identify disease-relevant Tregs in lung cancer and may be closely related to cancer malignancy, metastasis, and clinical outcomes. To test this possibility, we analyzed the percentage of GARP^+^ cells within the Foxp3^+^ Tregs from 39 tumor tissues and 50 PBs in lung cancer patients. As shown in Figure 1 and in Tables 1 and 2, although the proportion of total Tregs, as defined by Foxp3 expression, in the CD4 cells of tumor tissues [6.400% (4.780%, 13.20%)] was higher than that in PBs [5.890% (4.343%, 7.520%), *P* = 0.0439], no correlation was observed between the proportion of total Foxp3^+^ Tregs and the clinicopathological characteristics, such as gender (*P* = 0.0513 and *P* = 0.3657), age (*P* = 0.6737 and *P* = 0.2410), smoke (*P* = 0.0902 and *P* = 0.0631), histology (*P* = 0.9986 and *P* = 0.8211), lymphatic invasion (*P* > 0.9999 and *P* = 0.2566), distant metastasis (*P* = 0.1074 and *P* = 0.0737), and clinical stage (*P* = 0.7602 and *P* = 0.1153) in both tumor tissues and PBs.

**Figure 2:**
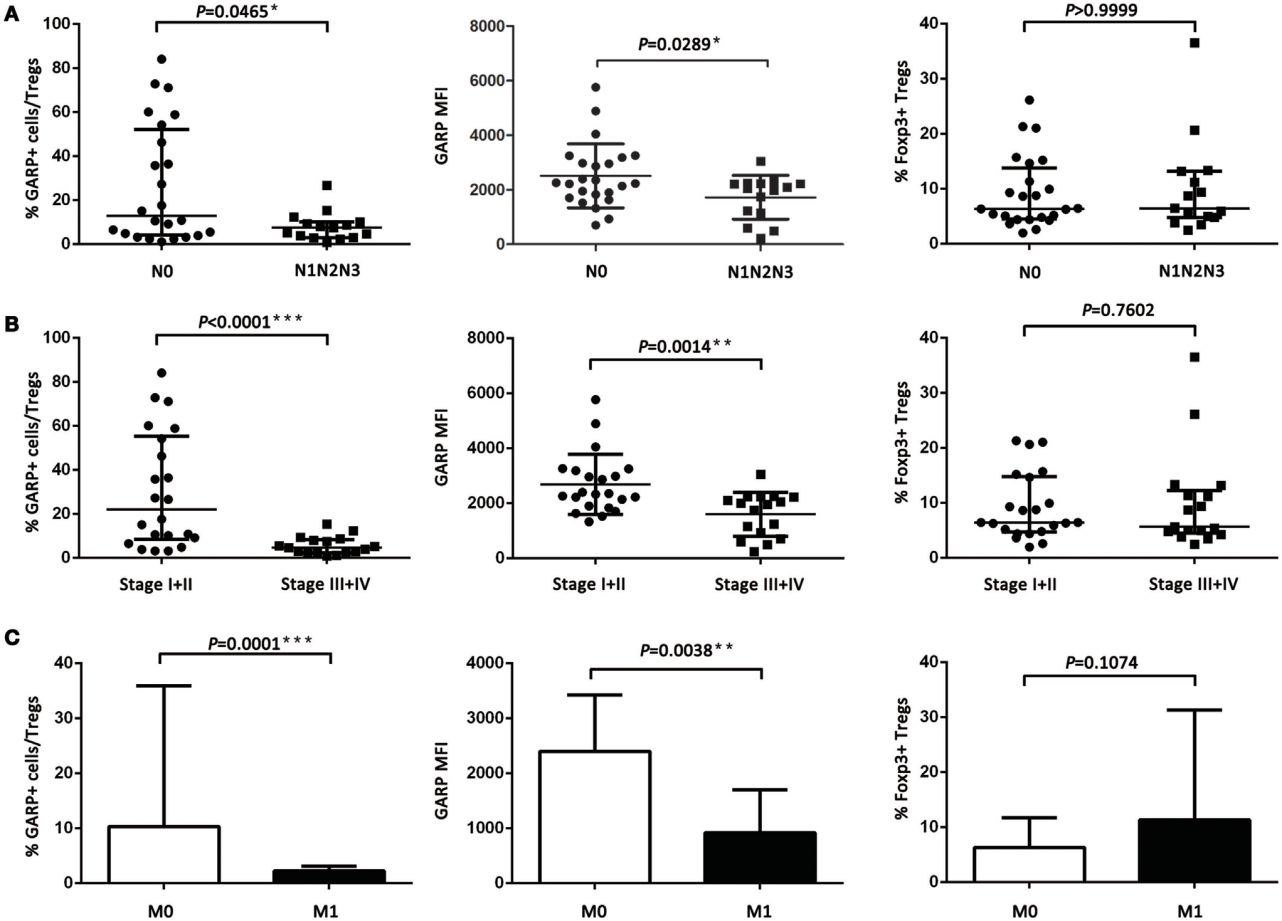
**Relationship between glycoprotein A repetitions predominant (GARP) expression in tumor tissues and clinicopathological characteristics of lung cancer patients**. **(A)** Proportion of GARP^+^ Tregs, mean fluorescence intensity (MFI) of GARP expression, and proportion of Foxp3^+^ Tregs in tumor tissues derived from patients without lymphatic invasion (N0, *N* = 24) and patients with lymphatic invasion (N1N2N3, *N* = 15). **(B)** Proportion of GARP^+^ Tregs, MFI of GARP expression, and proportion of Foxp3^+^ Tregs in tumor tissues from stage I (*N* = 10), stage II (*N* = 12), stage III (*N* = 12), and stage IV lung cancer patients (*N* = 5). **(C)** Proportion of GARP^+^ Tregs, MFI of GARP expression, and proportion of Foxp3^+^ Tregs in tumor tissues from lung cancer patients without distant metastasis (M0, *N* = 34) and lung cancer patients with distant metastasis (M1, *N* = 5). Results are expressed as mean ± SEM or median and interquartile range. *P* value shown is obtained from the comparison between the indicated groups by independent sample *t*-test and Kruskal–Wallis test.

However, the proportion of GARP^+^ subset in Tregs from lung cancer patients’ tumor tissues was highly correlated to lymphatic invasion (*P* = 0.0465), distant metastasis (*P* = 0.0001), and clinical stage (stage I + II vs. stage III + IV, *P* < 0.0001), whereas the proportion of GARP^+^ cells in Tregs was not correlated to gender (*P* = 0.3268), age (*P* = 0.9833), smoke (*P* = 0.1380), and histology (*P* = 0.6407) (Figure 2; Tables 1 and 2). Further, the MFI of GARP expression by Tregs was also highly correlated to lymphatic invasion (*P* = 0.0221), distant metastasis (*P* = 0.0038), and clinical stage (stage I + II vs. stage III + IV, *P* = 0.0014) (Figure 2; Tables 1 and 2). No association of the proportion of GARP^+^ cells and the MFI of GARP expression in Tregs from PBs with any clinicopathological characteristics of lung cancer patients was observed. Our data suggested that the proportion of GARP^+^ cells in Tregs from lung cancer patients without lymphatic invasion was higher than that from patients with lymphatic invasion [12.90% (4.090%, 52.13%) vs. 7.470% (2.920%, 10.10%)] while the MFI of GARP expression in Tregs was in accordance with GARP^+^ cell proportion (2,510 ± 1,176 vs. 1,724 ± 803.4) (Figure 2A). Tregs from lung cancer patients without distant metastasis exhibited more GARP expression than that from patients with distant metastasis [10.30% (5.005%, 35.88%) vs. 2.250% (0.9070%, 3.110%)] (2,398 ± 1,023 vs. 916.2 ± 779.2) (Figure 2C). In addition, Tregs from the patients in stages I and II expressed more GARP than that from patients in stages III and IV (Figure 2B). Therefore, these data indicate that the expression levels of GARP by Tregs were highly associated with clinical pathology, and thus may prove to be useful as a prognostic biomarker at the early stage of lung cancer.

### Effect of Cancer Cells on the GARP Expression in Tregs

**Figure 3:**
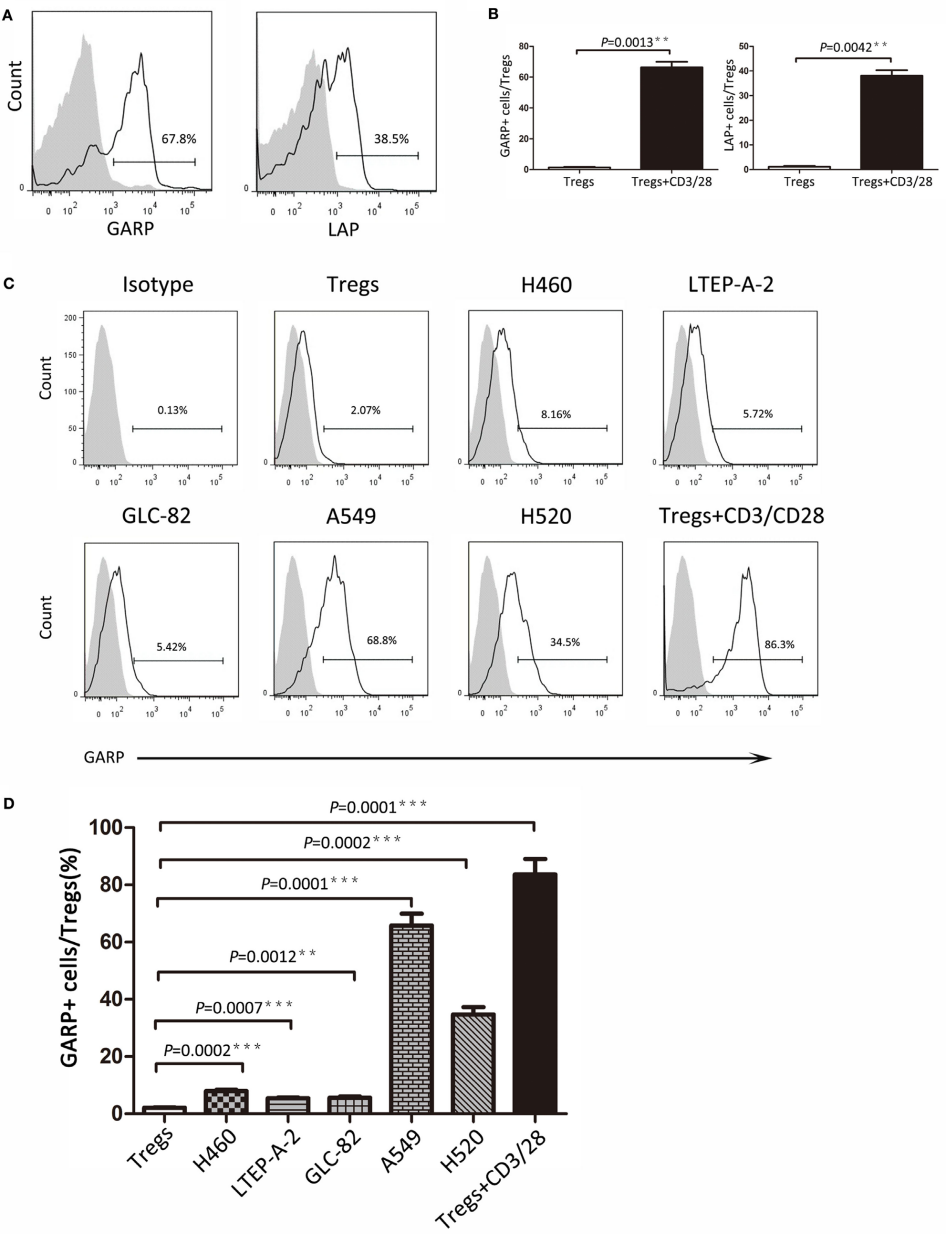
**Continued Different lung cancer cell lines induced glycoprotein A repetitions predominant (GARP) expression on Tregs from peripheral bloods (PBs) of lung cancer patients**. CD4^+^CD25^+^ Tregs were isolated from the PBs of lung cancer patients. After 72 h of TCR stimulation, these subsets were stained with CD4, GARP, latency-associated peptide (LAP), and intracellular Foxp3 and analyzed with FACS. **(A)** Typical FACS plots. Black histogram: Ab staining after TCR stimulation; gray-filled histogram: negative control (before TCR stimulation). Numbers in the plots indicate the proportion of positive cells after TCR stimulation. **(B)** Summary of proportion of GARP^+^ and LAP^+^ Tregs before and after TCR stimulation. Coculture assays were performed between lung cancer cell lines and CD4^+^CD25^+^ Tregs, which were isolated from the PBs of lung cancer patients for 72 h. Tregs were stained for CD4, GARP, and intracellular Foxp3 and analyzed with FACS. **(C)** Typical FACS plots. Black histogram: Ab staining; gray-filled histogram: isotype control. Numbers in the plots indicate the proportion of positive cells. **(D)** Summary of the proportion of GARP^+^ Tregs cocultured with different lung cancer cell lines. Results are expressed as mean ± SEM (*n* = 3). *P* value shown is obtained from the comparison between the indicated groups by two-sample *t*-test.

As we know, GARP can bind latent TGF-β formed by mature TGF-β and LAP to mediate the suppressive function of Tregs. In this study, we detected the GARP and LAP expression after TCR stimulation using CD3/CD28 dynabeads by flow cytometry. Tregs were isolated from lung cancer patient PBs and cultured with CD3/CD28 dynabeads for 48 h. According to results, the proportion of GARP^+^ cells in Foxp3^+^ Tregs was notably increased after TCR stimulation (66.33 ± 4.067% vs. 1.333 ± 0.4333%, *P* = 0.0013) (Figures 3A,B). The LAP expression in Foxp3^+^ Tregs after TCR stimulation was also higher than that in the negative control (38.07 ± 4.267% vs. 1.1 ± 0.2001%, *P* = 0.0042) (Figures 3A,B). In conclusion, TCR stimulation in Tregs induces the GARP expression, which further leads to LAP expression and mediation of TGF-β secretion.

Glycoprotein A repetitions predominant may be a biomarker at the early stage of lung cancer and may also mediate the suppressive function of Tregs. Thus, further studies are need to determine whether cancer cells have influence on GARP expression in Tregs and trigger the suppressive function of Tregs mediated by GARP, leading to immunosuppression and tumor escape. We designed the coculture assays using the Tregs isolated from lung cancer patients PBs with lung cancer cell lines (H460, LTEP-A-2, GLC-82, A549, and H520) for 72 h. Flow cytometry results indicated that the proportions of GARP^+^ cells in Foxp3^+^ Tregs were 7.960 ± 0.3940%, 5.427 ± 0.3060%, and 5.640 ± 0.3911% when cocultured with H460, LTEP-A-2, and GLC-82, respectively, which were slightly higher than that in the negative control (2.090 ± 0.1735%) (Figures 3C,D). In sharp contrast, A549 and H520 can effectively induce GARP expression in Tregs. The proportion of GARP^+^ cells reached 65.80 ± 4.149% and 34.73 ± 2.578%, which were far higher than that in other lung cancer cell lines coculture assays, whereas that in the positive control was 83.60 ± 5.453% (Figures 3C,D). According to these data, some certain lung cancer cell lines can promote Tregs to express GARP. Although the abilities of lung cancer cell lines to induce GARP expression were different from each other, some lung cancer cell lines, such as A549 and H520, could enhance the suppressive function of Tregs through high expression of GARP.

### Lung Cancer Cell Lines Induced GARP Expression in Tregs

**Figure 4:**
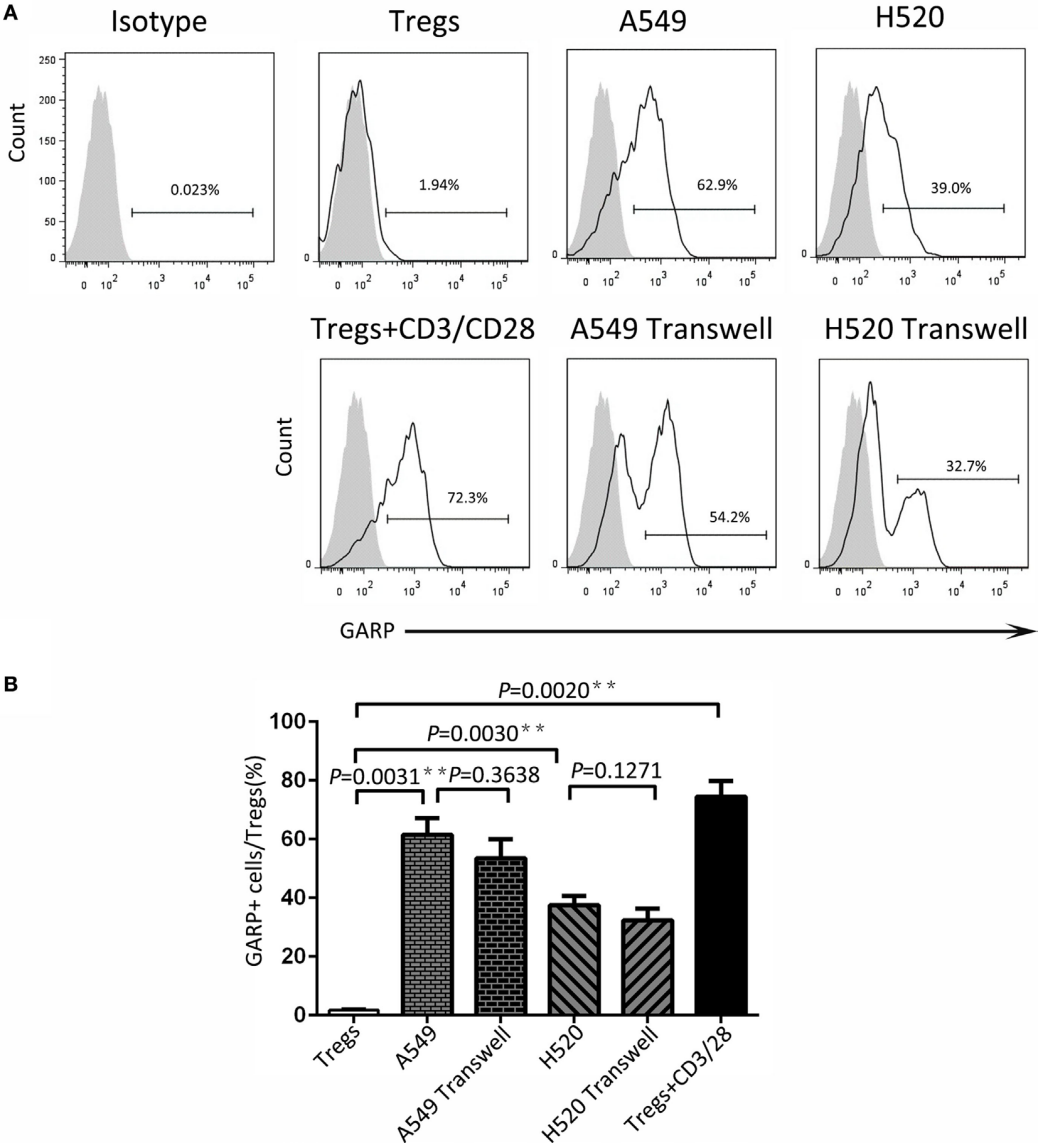
**Level of glycoprotein A repetitions predominant (GARP) expression on Tregs by transwell coculture assays**. Transwell coculture assays were performed between lung cancer cell lines (A549 and H520) and CD4^+^CD25^+^ Tregs, which were isolated from peripheral bloods of lung cancer patients for 72 h. Tregs were stained for CD4, GARP, and intracellular Foxp3 and analyzed with FACS. **(A)** Typical FACS plots. Black histogram: Ab staining; gray-filled histogram: isotype control. Numbers in the plots indicate the proportion of positive cells. **(B)** Summary of the proportion of GARP^+^ Tregs in Transwell and contact cocultured groups with A549 and H520, respectively. Results are expressed as mean ± SEM (*n* = 3). *P* value shown is obtained from the comparison between the indicated groups by two-sample *t*-test.

**Figure 5:**
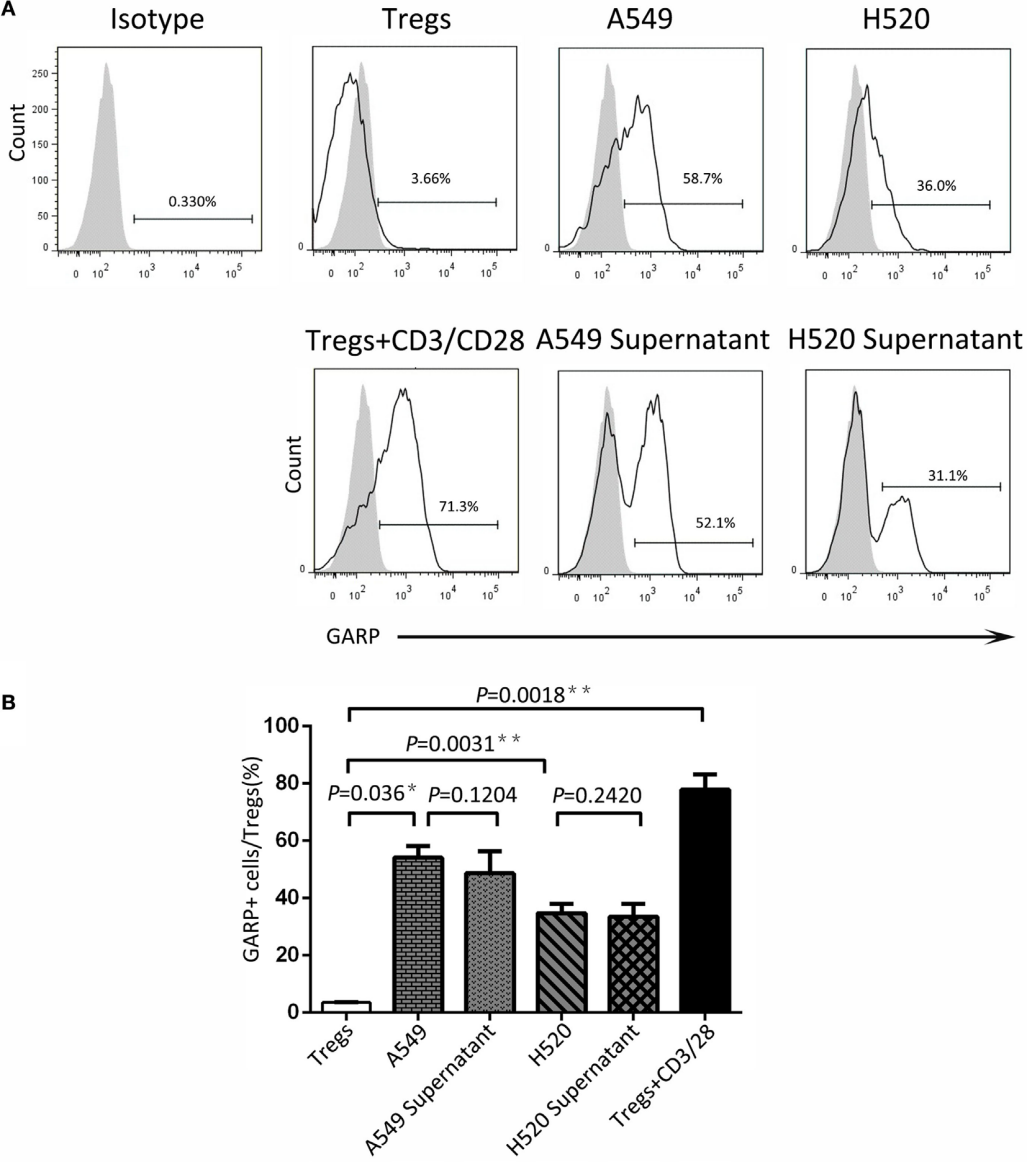
**Cancer cell line supernatant induced glycoprotein A repetitions predominant (GARP) expression on Tregs**. The supernatant of cancer cell lines (A549 and H520) was collected to culture CD4^+^CD25^+^ Tregs, which were isolated from the peripheral bloods of lung cancer patients for 72 h. Tregs were stained with CD4, GARP, and intracellular Foxp3 and analyzed with FACS. **(A)** Typical FACS plots. Black histogram: Ab staining; gray-filled histogram: isotype control. Numbers in the plots indicate the proportion of positive cells. **(B)** Summary of the proportion of GARP^+^ Tregs in supernatant cultured and contact cocultured groups with A549 and H520, respectively. Results are expressed as mean ± SEM (*n* = 3). *P* value shown is obtained from the comparison between the indicated groups by two-sample *t*-test.

To seek the possible mechanism of lung cancer cell lines inducing GARP expression in Tregs, we used A549 and H520, which could effectively promote Tregs to express high levels of GARP to do the transwell coculture assays. After 72 h of coculturing, the proportions of GARP^+^ subsets in Foxp3^+^ Tregs from A549 transwell and contact coculture groups were nearly the same (53.53 ± 6.417% vs. 61.57 ± 5.525%, *P* = 0.3638) whereas that of the negative control was 1.777 ± 0.2314% and that of the positive control was 74.50 ± 5.368% (Figure 4). Similar to A549 groups, the proportions of GARP^+^ cells from H520 transwell and contact coculture groups were also the same (32.40 ± 6.710% vs. 37.57 ± 3.171%, *P* = 0.1271) (Figure 4). These demonstrated that cell contact was not the mode of interaction between Tregs and lung cancer cell lines. Therefore, we speculated that cancer cells can secrete some cytokines to induce GARP expression in Tregs. To test this hypothesis, we collected the cell supernatant of cancer cells A549 and H520. Then we used these supernatant to culture Tregs isolated from lung cancer patients PBs for 72 h. The proportions of GARP^+^ cells in Foxp3^+^ Tregs from A549 supernatant and contact culture groups were 48.53 ± 7.681% and 54.03 ± 4.038%, respectively, whereas that from the H520 supernatant and contact culture groups were 33.430 ± 4.485% and 34.60 ± 3.252%, respectively (Figure 5). Our data suggested that cancer cells induced GARP expression in Tregs through other ways instead of cell contact, such as secreted cytokines. Further studies are needed to confirm this hypothesis.

## Discussion

Tregs as the main immunosuppressive cells play an important role in immunity regulation, which is also associated with tumor progression. Many molecules contribute to the immunosuppressive function of Tregs, such as cytotoxic T lymphocyte-associated antigen 4 (CTLA-4), tumor necrosis factor receptor 2, lymphocyte activation gene 3, T cell membrane protein 3, and GARP (26–29). The exact mechanism of Treg immunosuppressive function is unclear, and the definite roles of these molecules in the regulatory effect are also unknown. One sure thing was that GARP^+^ Tregs possessed high immune suppression, and GARP–TGF-β pathway is the major way of suppressive function in this subset.

Previous studies have reported that higher population of Tregs was present in lung cancer patients than that in healthy donors (30, 31). Our previous work (28) has demonstrated that and this studies further reveal that the numbers of Tregs from tumor tissues were also higher than that from PBs in lung cancer patients (Figure 1E). However, we did not find the correlation between the levels of total Foxp3^+^ Tregs, neither from tumor tissues or PBs with clinicopathological characteristics of the patients, which was in agreement with our previous the studies and other previous reports (28, 32, 33). GARP expression level was significantly increased on tumor-infiltrating Tregs compared with that on PBs (Figure 1F). Interestingly, our data indicated that Tregs from the tumor tissues of lung cancer patients without lymphatic invasion or distant metastasis expressed more GARP than those from lung cancer patients with lymphatic invasion or distant metastasis, and Tregs from patients in stages I and II also had more GARP expression than those from patients in stages III and IV. No correlation was observed between the GARP expression level on Tregs from PBs with the clinicopathological characteristics of the patients (Tables 1 and 2). Therefore, GARP might be an early prognostic biomarker because it is specifically expressed on the surface of tumor-infiltrating Tregs, and lung cancer patients with early stage had more GARP^+^ Tregs than advanced cancer patients. As a result of the complex and unclear regulatory network of the molecules, which contributed to the Tregs immunosuppressive function, different molecules may play an important role at different stages of cancer. According to our study, GARP, which endowed Tregs immunosuppression by GARP–TGF-β pathway, may possess a key role in the early stage of cancer. Another reason to support our hypothesis is that TGF-β could play a critical role for the function of Tregs, which may inhibit Teffs proliferation and cytokines production as well as promote the differentiation of Teffs into Tregs (34, 35).

Further, we designed *in vitro* coculture assays using lung cancer cell lines and Tregs from patients’ PBs to verify that cancer cells can induce Tregs GARP expression, which may confer and launch the Treg immunosuppression at the early stage of cancer. Our study demonstrated that lung cancer cells could induce GARP expression on Tregs by secreting some certain cytokines, which need to be identified. The ability of different cell lines to stimulate the GARP expression were not identical, for example, A549 and H520 could induce high GARP expression on Tregs whereas H460, LTEP-A-2, and GLC-82 can only cause rather few GARP expression. These assays supported our hypothesis that GARP might be an early prognostic biomarker. In the tumor microenvironment, tumor cells induced tumor-infiltrating Tregs to express GARP. On the one hand, GARP endowed Treg immune suppression to inhibit effector T cell. On the other hand, GARP played an important role in TGF-β enrichment and release on Treg surfaces, which was negative regulatory factor and could promote Teffs differentiation into Tregs. Therefore, tumor cells could escape from the immune system by inducing GARP expression on Tregs to suppress Teffs in tumor microenvironment and lead to cancer progression.

The discovery of GARP on Tregs sets a new stage in elucidating functions and mechanism of Tregs. Although some studies indicated that GARP was not absolutely required for the suppressive function of Tregs (36), our study indicated that GARP was critical in initiating Treg activation, and it might participate in Treg suppressive function. The GARP–TGF-β pathway provides a regulatory network between Tregs and their targets, including Tregs themselves. We also should notice that the regulatory function mediated by GARP in Tregs is not the whole but just a part. Many other molecules, such as CTLA-4, are also reported to mediate Treg suppressive function. Until now, our understanding of GARP and the relationships of these molecules are limited and are unclear. Clear elucidation is a difficult challenge in the future. Further studies may confirm that GARP is a crucial molecule for Tregs not only for identification but also for tumor immunotherapy.

## Ethics Statement

This study was carried out in accordance with the recommendations of the ethical standards of the Institutional Review Committee on Human Research of the Tianjin Medical University Cancer Institute and Hospital with written informed consent from all subjects. All subjects gave written informed consent in accordance with the Declaration of Helsinki. The protocol was approved by the Institutional Review Committee on Human Research of the Tianjin Medical University Cancer Institute and Hospital.

## Author Contributions

HL and HJ conceived the study, analyzed the data, and wrote the manuscript. HJ, LS, LT, and WY performed the research. All the authors read and approved the final manuscript.

## Conflict of Interest Statement

The authors declare that the research was conducted in the absence of any commercial or financial relationships that could be construed as a potential conflict of interest.

## Supplementary Material

Figure S1 **Relationship between glycoprotein A repetitions predominant (GARP) expression in peripheral bloods (PBs) and clinicopathological characteristics of lung cancer patients**. **(A)** Proportion of GARP^+^ Tregs, MFI of GARP expression, and proportion of Foxp3^+^ Tregs in PBs derived from patients without lymphatic invasion (N0, *N* = 24) and patients with lymphatic invasion (N1N2N3, *N* = 26). **(B)** Proportion of GARP^+^ Tregs, mean fluorescence intensity (MFI) of GARP expression, and proportion of Foxp3^+^ Tregs in PBs from stage I (*N* = 15), stage II (*N* = 7), stage III (*N* = 20), and stage IV lung cancer patients (*N* = 8). **(C)** Proportion of GARP^+^ Tregs, MFI of GARP expression, and proportion of Foxp3^+^ Tregs in PBs from lung cancer patients without distant metastasis (M0, *N* = 42) and lung cancer patients with distant metastasis (M1, *N* = 8). Results are expressed as mean ± SEM or median and interquartile range. *P* value shown is obtained from the comparison between the indicated groups by Kruskal–Wallis test.
