# Supplementary figures and images for: Expression of GARP Is Increased in Tumor-Infiltrating Regulatory T Cells and Is Correlated to Clinicopathology of Lung Cancer Patients
Source: Front Immunol. 2017 Feb 14;8:138. doi: 10.3389/fimmu.2017.00138 (PMC5306210; doi:10.3389/fimmu.2017.00138)

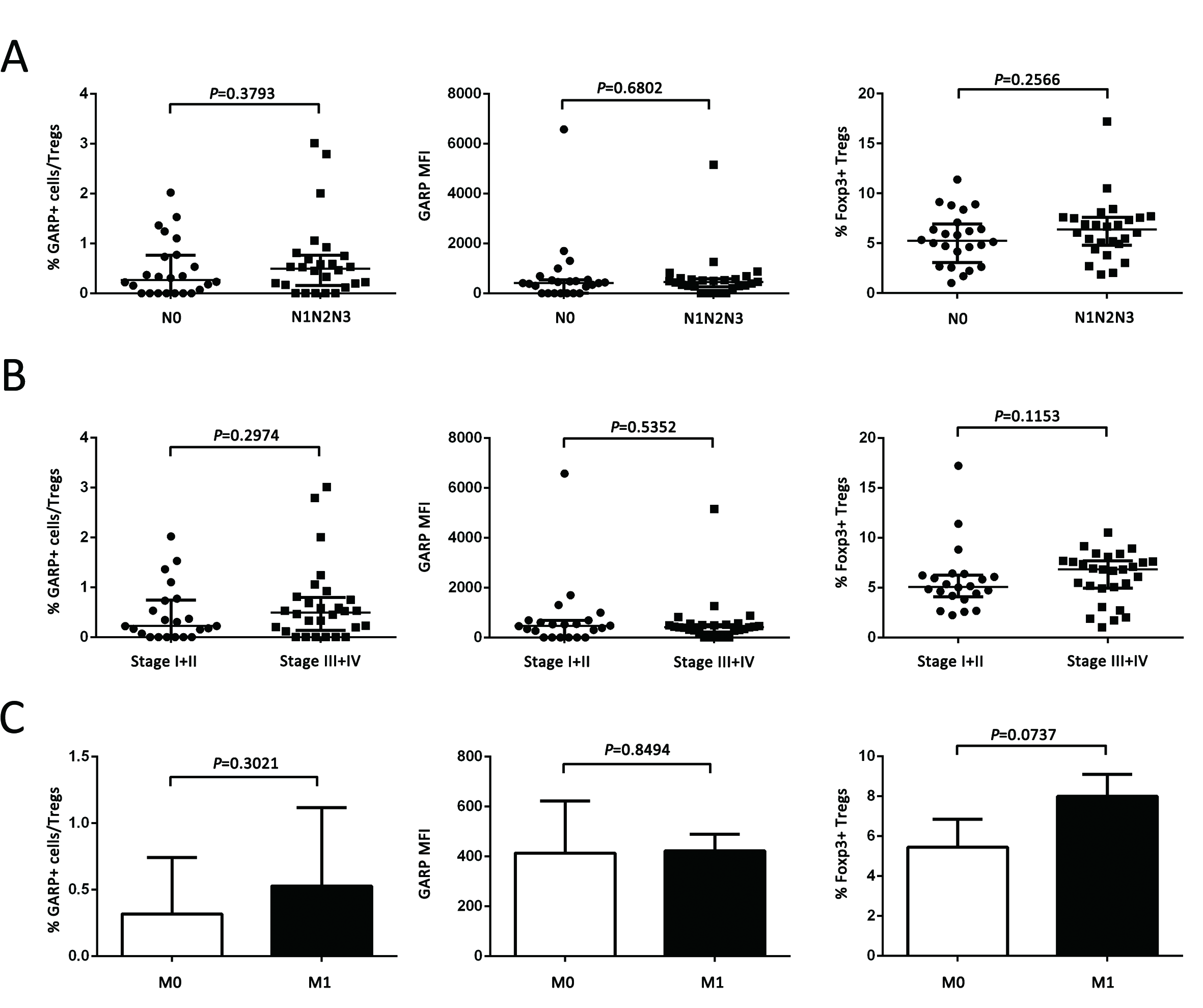

Supplement: Figure S1 — Relationship between glycoprotein A repetitions predominant (GARP) expression in peripheral bloods (PBs) and clinicopathological characteristics of lung cancer patients. (A) Proportion of GARP+ Tregs, MFI of GARP expression, and proportion of Foxp3+ Tregs in PBs derived from patients without lymphatic invasion (N0, N = 24) and patients with lymphatic invasion (N1N2N3, N = 26). (B) Proportion of GARP+ Tregs, mean fluorescence intensity (MFI) of GARP expression, and proportion of Foxp3+ Tregs in PBs from stage I (N = 15), stage II (N = 7), stage III (N = 20), and stage IV lung cancer patients (N = 8). (C) Proportion of GARP+ Tregs, MFI of GARP expression, and proportion of Foxp3+ Tregs in PBs from lung cancer patients without distant metastasis (M0, N = 42) and lung cancer patients with distant metastasis (M1, N = 8). Results are expressed as mean ± SEM or median and interquartile range. P value shown is obtained from the comparison between the indicated groups by Kruskal–Wallis test. [file image_1.tif]
